# Recognition of Sheep Feeding Behavior in Sheepfolds Using Fusion Spectrogram Depth Features and Acoustic Features

**DOI:** 10.3390/ani14223267

**Published:** 2024-11-13

**Authors:** Youxin Yu, Wenbo Zhu, Xiaoli Ma, Jialei Du, Yu Liu, Linhui Gan, Xiaoping An, Honghui Li, Buyu Wang, Xueliang Fu

**Affiliations:** 1 College of Computer and Information Engineering, Inner Mongolia Agricultural University, Hohhot 010018, China; yxyu1@emails.imau.edu.cn (Y.Y.); zhuwbcs@emails.imau.edu.cn (W.Z.); maxiaoli@emails.imau.edu.cn (X.M.); dujialei@emails.imau.edu.cn (J.D.); liuyu598850462@emails.imau.edu.cn (Y.L.); ganlh@emails.imau.edu.cn (L.G.); lihh@imau.edu.cn (H.L.); 2 Key Laboratory of Smart Animal Husbandry at Universities of Inner Mongolia Autonomous Region, Inner Mongolia Agricultural University, Hohhot 010018, China; anxiaoping@imau.edu.cn; 3 College of Animal Science, Inner Mongolia Agricultural University, Hohhot 010018, China

**Keywords:** sheep feeding behavior, spectrogram, SheepVGG-Lite, deep learning, feature fusion

## Abstract

In precision feeding, non-contact and pressure-free monitoring of sheep feeding behavior is crucial for health monitoring and optimizing production management. The experimental conditions and real-world environments differ when using acoustic sensors to identify sheep feeding behaviors, leading to discrepancies and consequently posing challenges for achieving high-accuracy classification in complex production environments. This study enhances the classification performance by integrating the deep spectrogram features and acoustic characteristics associated with feeding behavior. We conducted the task of collecting sound data in actual production environments, considering noise and complex surroundings. The method included evaluating and filtering the optimal acoustic features, utilizing a customized convolutional neural network (SheepVGG-Lite) to extract Short-Time Fourier Transform (STFT) spectrograms and Constant Q Transform (CQT) spectrograms’ deep features, employing cross-spectrogram feature fusion and assessing classification performance through a support vector machine (SVM). Results indicate that the fusion of cross-spectral features significantly improved classification performance, achieving a classification accuracy of 96.47%. These findings highlight the value of integrating acoustic features with spectrogram deep features for accurately recognizing sheep feeding behavior.

## Simple Summary

Precision feeding requires reliable methods to monitor the feeding behavior of sheep. It is especially critical to achieve a high-accuracy classification of sheep feeding behavior in complex environments through acoustic sensors. This study collected data from production environments and thoroughly considered noise interference. The feature fusion technique significantly improved the recognition performance. The results show that combining multiple features makes the classification accuracy reach 96.47%. This technique can automatically monitor the feeding behavior of sheep, which helps to improve breeding efficiency and animal welfare and significantly promotes the development of intelligent sheep farming.

## 1. Introduction

Monitoring of non-contact, stress-free eating behavior in sheep plays a crucial role in precision feeding [1]. Accurate identification of feeding behavior in sheep is essential for health monitoring and enables the optimization of feed formulation and feeding strategies. The feeding behavior of sheep is divided into two categories: foraging and ruminating [2]. Foraging is the behavior of sheep to actively ingest food, which directly affects their growth, development, and health status. Feeding frequency and duration reflect sheep health and appetite, so real-time monitoring helps detect diseases and improve feeding management. Rumination is the process by which sheep return swallowed food from the stomach to the mouth for re-chewing, which helps to break down crude fiber further and promote nutrient absorption. Changes in rumination behavior may be an early warning signal of digestive abnormalities. Therefore, monitoring rumination behavior is essential for assessing the digestive health of sheep and preventing gastrointestinal diseases.

However, relying on the manual inspections conducted by on-site veterinarians may not only lead to delayed responses but also significantly increase costs, with the results often bearing subjective biases. To address these issues, researchers are developing automated detection technologies to identify sheep grazing and rumination accurately. These advancements in automated technologies have vastly improved detection accuracy and real-time responsiveness while also facilitating efficient and thorough behavior monitoring in large-scale farming settings, ultimately supporting the progress of intelligent farming initiatives. The automated detection system can promptly identify abnormal behaviors, prompting farmers to take timely intervention measures, thereby optimizing farm management and enhancing production efficiency. This technological application will significantly reduce reliance on the workforce, lower costs, and, based on data-driven foundations, provide more objective health assessments and management decisions.

In agricultural IoT, sensor devices have been widely applied to enhance the capabilities for remote, extensive, and large-scale monitoring of flock behavior [1]. According to the data sources of sensors, the existing literature can be roughly divided into two categories: non-contact sensors and contact sensors. Non-contact sensors primarily include cameras [1]. While these sensors can be used to identify flock behavior, their accuracy is somewhat limited compared with contact devices [3,4]. Visual sensors in sheep can be further used to combine individual recognition with feeding behavior recognition [5], enabling more accurate management and monitoring. Contact sensors primarily include motion sensors and acoustic sensors. While motion sensors detect general movement, acoustic features capture the specific sound patterns associated with feeding, offering a more direct measure of feeding behavior characteristics. Compared with motion sensors, acoustic sensors are less sensitive to location [6], making them particularly suitable for monitoring sheep behavior in confined environments. Traditional machine learning methods often rely on manually designed acoustic features to identify sheep feeding behaviors, combined with classifiers such as support vector machines, random forests, or linear discriminant analysis. While effective in specific conditions, these methods rely on manual feature selection, limiting their ability to capture complex patterns [7,8].

To overcome the limitations of traditional methods, some researchers have begun to explore the use of more advanced deep learning models, such as long short-term memory networks, to process acoustic signals. This approach handles time series data better and enhances classification performance [9]. This enhancement has made significant strides in capturing the temporal patterns of sheep behavior. Nevertheless, despite the ability of deep learning models to independently acquire certain features [10,11], they remain, to some extent, dependent on manual feature extraction, which restricts their efficacy in the realm of fully automated behavior recognition.

With the continuous advancement of computational power, recent research has gradually shifted towards more automated feature extraction methods. Some researchers attempt to use spectrograms as input, leveraging convolutional neural networks to extract deep features [12,13]. Convolutional neural networks automatically learn multi-level features, reducing reliance on manual input. This ability allows them to capture the time–frequency characteristics of sheep behavior more effectively. To further enhance classification performance, some studies have integrated recurrent neural networks to model temporal information, forming a framework that combines convolutional neural networks and recurrent neural networks to predict sheep behavior with greater precision [12]. Despite the progress made by these methods, two pivotal challenges persist in the study of identifying sheep feeding behavior using acoustic sensors.

First and foremost, the disparity between experimental conditions and actual production environments constitutes a significant issue [12,13]. In numerous studies, the acoustic data of sheep feeding behavior are typically collected in highly controlled experimental environments, which are relatively tranquil and subject to minimal external interference. While idealized recording conditions can effectively reduce noise interference and thereby improve the accuracy of the model’s classification, they overlook the complexities present in real-world farming environments. In real-world production environments, farms are typically permeated with a myriad of noises from other animals, machinery, and the natural environment, such as wind and rain. These intricate acoustic backgrounds may obscure the sounds of sheep grazing and interfere with the model’s feature extraction process, leading to diminished classification performance. These lab-to-field data discrepancies reduce model performance in real-world applications, limiting its practical use on farms.

Another critical issue lies in achieving high-accuracy classification in complex environments. Even though the model demonstrates high accuracy under experimental conditions, it remains challenging to achieve consistent classification results when applied to actual farms. The primary challenge is primarily due to the extensive and unpredictable sources of noise in production environments, which can easily interfere with the model, significantly reducing the accuracy of feeding behavior recognition. In the context of real-world farm environments, challenges such as background noise from other animals (e.g., birds and dogs), weather-related sounds (e.g., wind and rain), and the dynamic interactions among freely moving sheep introduce significant complexity to the accurate monitoring of sheep feeding behavior. Particularly in communal rearing environments, the vocalizations of multiple individuals often overlap, rendering the precise isolation of the feeding sounds of the target sheep an arduous task. Moreover, the feeding behavior of sheep is inherently diverse and dynamic, with significant variations in the feeding patterns of different individuals, further complicating the model’s recognition process. These factors make acoustic-based models less robust and effective in complex farm environments.

Drawing on the results of Wang et al., who enhanced the accuracy of pig cough recognition by integrating deep features with acoustic features [14,15], we address two critical issues in the identification of sheep feeding behavior. Inspired by this revelation, we plan to enhance sheep feeding behavior’s classification accuracy by integrating deep spectrogram features with acoustic characteristics. Unlike previous studies that collected data under ideal experimental conditions, this research gathered data in a natural production environment, fully accounting for the impact of noise and complex situations in the farming context. This data collection method effectively addresses the challenges present in current research, specifically the decrease in classification accuracy of existing models in complex environments. By using real-world data, we aim to improve model robustness for stable performance in production settings.

This study aims to capture distinct time–frequency patterns in sheep feeding sounds by integrating acoustic and spectrogram deep features derived from the housing environment. In pursuit of this, the goal is to establish a resilient and efficient feature representation that significantly enhances the accuracy of feeding behavior recognition. To achieve this, we proposed a method that first evaluates the acoustic features and selects an optimal subset to ensure their comprehensive utilization. Secondly, we employed the fully connected layers of a convolutional neural network to extract MEL spectrograms, Short-Time Fourier Transform spectrograms, and Constant Q Transform spectrograms’ deep features and evaluate the recognition performance of these deep features through a support vector machine (SVM). Third, shallow convolutional neural networks (CNNs) were compared with fine-tuned models pre-trained on ImageNet to evaluate their recognition accuracy, feature extraction speed, and model size, aiming to identify the most suitable model architecture. Finally, using feature fusion, optimal deep and acoustic features were combined to improve recognition accuracy.

This research has made several key contributions:

1. Development of a novel framework: We present a new approach that combines acoustic features with deep characteristics to recognize sheep feeding behavior.
2. Developed an innovative convolutional neural network: We built a carefully designed architecture to extract deep features from spectrograms, improving the accuracy of sheep feeding behavior recognition.
3. Conducted a comparative analysis with existing models: The method introduced in this study demonstrates superior performance over current CNN models, particularly in recognition efficiency, model compactness, and classification accuracy.
4. Model Interpretability Analysis: Using EigenCAM visualization techniques, the model’s feature activations for behaviors such as sheep feeding, rumination, and bleating were successfully demonstrated, thereby significantly enhancing the interpretability and accuracy of behavior classification.

The rest of this paper is organized as follows: the materials section explores the data used in the experiment and related prior work. The methodology section comprehensively describes the methods used in the experiment. The results section presents the findings obtained from our experiment. The discussion section provides an in-depth analysis of these results. Finally, the conclusion summarizes the main findings and contributions.

## 2. Materials and Methods

### 2.1. Housing and Laboratory Animals

**Figure 1:**
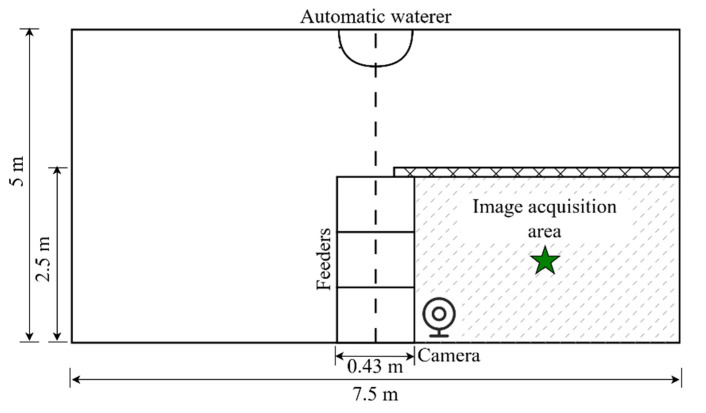
Diagrammatic representation of the experimental sheep enclosure. The star indicates the center of the image acquisition area.

The data for this study were sourced from a sheep barn at a small commercial farm located in Hohhot, Inner Mongolia Autonomous Region, China. The data were collected in an actual production environment. In 2024, at the end of May, the stock of sheep on this farm was 45 heads, including breeds such as Texel, Suffolk, and Dorper. Within this experiment, we selected sheep (aged 1 to 3 years) as our study subjects, comprising one Texel sheep, one White Suffolk sheep, and four Dorper sheep. The sheep roamed freely within the experimental barn. The specific layout of the experimental sheep pen is depicted in Figure 1. The sheep pen was equipped with water facilities available throughout the day, with drinking water stored in troughs, and alfalfa hay was supplied in the central feeding trough for the sheep to consume all day. The upper part of the sheep pen was equipped with a roof.

### 2.2. Data Collection and Preprocessing

**Figure 2:**
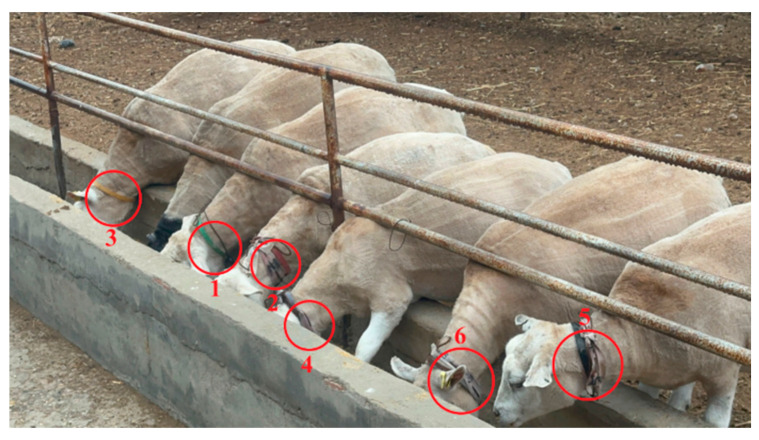
Arrangement of recording apparatus on sheep. Each sheep is numbered to correspond with the recording.

The data collection system of this study was based on the actual production environment deployed in the experimental sheep pen, utilizing a cloud-edge architecture for data transmission and processing. In this experiment, the terminal equipment included a wireless microphone for collecting sound data and spherical cameras deployed in the sheep pen. The camera model was Keda, with a resolution of 1920 × 1080 and a frame rate of 25 frames per second, utilizing H.264 encoding format. The camera was fixed on one side of the sheep pen at a height of 2.5 m on the roof, inclined downwards, covering the entire area of the sheep pen to ensure comprehensive monitoring. Each experimental sheep was equipped with a wireless audio sensor on its neck (sampling rate of 48,000 Hz) (Figure 2) to record their respective sound data. The camera and microphone automatically collected audio and video data from the sheepfold during the preset periods, thereby providing the necessary information for subsequent behavior recognition.

The edge server was deployed in the server room of the breeding farm, utilizing a desktop PC server (OptiPlex 3070, Dell, Round Rock, TX, USA), achieving seamless connectivity with cameras and microphones via Ethernet. The edge server utilized FFMPEG (3.2.19) software to capture real-time video and audio data from the visible light channel (RTSP) with a resolution of 1920 × 1080. The collected video and audio streams were saved to the server’s hard drive in real time. Video files were named according to the start time of the recording, following the format “StartTime_CameraNumber.mp4”, with a new MP4 file generated every 60 min. Audio files were also named based on the start time of the recording, using the format “StartTime_MicrophoneNumber.wav”, with a new WAV file generated every 60 min. In this manner, the system could efficiently store and manage experimental data, ensuring subsequent data processing and analysis continuity and integrity.

This study utilized an internet connection to synchronize data between the edge and storage servers within the cloud-based animal husbandry platform. It utilized the Rsync file synchronization protocol for automatic and incremental file transfers. After each transmission, the edge server deleted files to optimize synchronization and storage. The data collection system of this study has practical production applications. It possesses excellent end-to-end generalization capabilities and can operate efficiently in complex farm environments [16].

**Figure 3:**
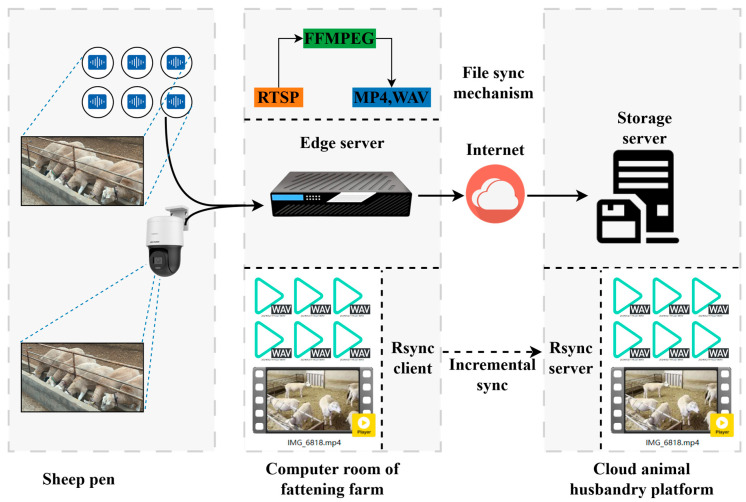
Architecture of the data acquisition and synchronization system.

Through the cloud-edge data collection system under the cloud animal husbandry platform (Figure 3), during the 7-day experiment period, data were collected from 6 sheep, including 44 h of audio recordings and corresponding visible light video data. Figure 2 presents the architecture of the data acquisition system, showing the complete workflow from the terminal devices to the cloud platform.

This study used Audacity (3.6.1) software to extract specific audio segments from the WAV files. Audio data annotation was divided into two parts: the first part involved behaviors that could be directly annotated based on visible light video, such as feeding behaviors (including foraging and rumination) and drinking behaviors; the second part required annotation through listening to audio data, such as sheep vocalizations, panting, rumination coughing, and other environmental noises (including sounds from other animals like birds and dogs, friction sounds between equipment and troughs, and background sounds from wind and rain).

Experts meticulously annotated each audio file during the operational process by referencing the sheep’s behavior observed in the visible light video. The annotations encompassed three primary behavioral tags: foraging on hay, ruminating, and drinking water. All these behaviors could be distinctly identified through visible light video. During this annotation process, we meticulously documented the start and end points of each audio segment’s corresponding behavior, thereby precisely delineating the audio fragments associated with these behaviors. In addition, to avoid interference from echoes or sounds of neighboring sheep, if the observed sheep did not exhibit the appropriate behavior, we considered the sound from a physically close sheep as noise and did not label it, even if the microphone captured the sound from a physically close sheep. Multiple sheep voices did overlap during the recording process, but we did not process them for additional separation. Given that the goal of this study was to achieve accurate feeding management, these sound overlaps reflected a common scenario in real feeding environments. Therefore, retaining these overlapping sound data provides a more accurate representation of sound characteristics in real-world environments.

Subsequently, the behavior-related segments already marked were removed from the original audio file to annotate other sounds. Subsequently, we utilized Python scripts to divide the remaining audio files into separate segments. The segmentation process necessitates the configuration of two pivotal parameters: the energy threshold and the time threshold. Upon completion of segmentation, these independent audio segments undergo cluster analysis. A veterinarian manually annotated each segment, identifying various sounds. The annotated sound types included sheep bleats, panting, rumination coughs, and other ambient noises.

Ultimately, through the steps above, we have acquired two categories of sound data: one pertains directly to the behaviors of sheep, representing specific actions such as feeding, ruminating, and drinking; the other encompasses various sounds within the breeding environment, including ambient noise.

### 2.3. Dataset

**Figure 4:**
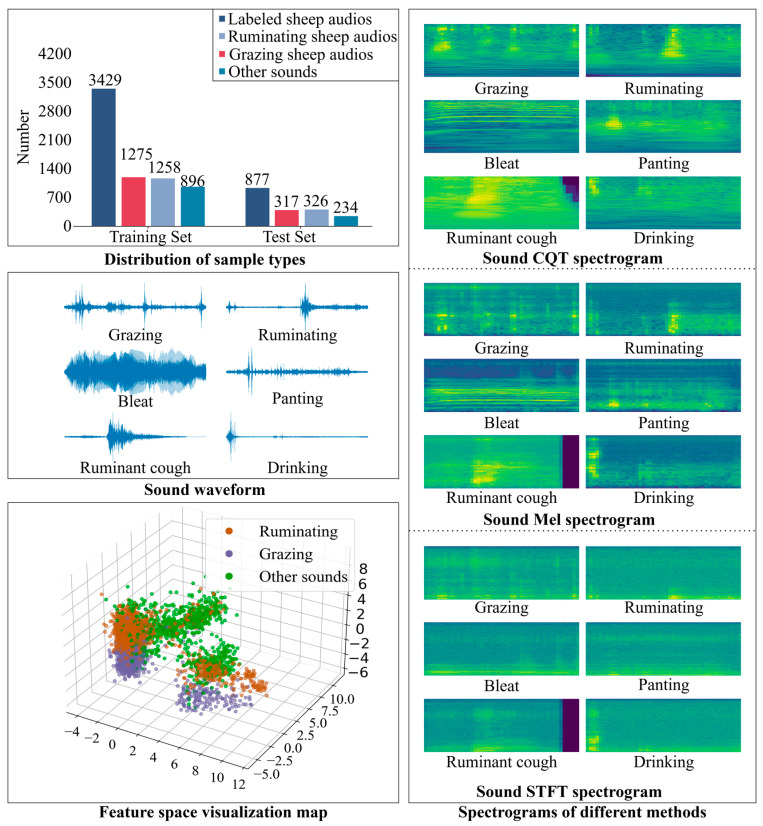
The distribution, spectral analysis, and feature visualization of sheep behavior audio data.

We used the audio files from the data collection and preprocessing phase to create the experimental dataset. Specifically, we selected 1592 audio clips of sheep feeding, 1584 audio clips of rumination, and 1130 audio clips of other sounds (including other behaviors of the sheep and other ambient noises), as shown in Figure 4.

To gain a profound understanding of the characteristics of these audio signals, we first extracted their waveform information, uncovering the sound patterns associated with different behaviors. The waveform of grazing behavior exhibits precise regularity, while the sound rhythm of rumination behavior is relatively slow and sustained. In contrast, the waveforms of actions such as sheep bleating and coughing appear more intense and erratic.

Additionally, we employed three distinct spectrum analysis methods (CQT spectrogram, Mel spectrogram, STFT spectrogram) to deeply explore the time–frequency characteristics of these audio recordings. The detailed explanations of the CQT spectrogram, Mel spectrogram, and STFT spectrogram can be found in Section 2.4.2. Through a comparison of various techniques, we determined that the CQT spectrograms were highly effective in capturing the critical features of sheep feeding and rumination behaviors. These behaviors exhibited relatively continuous and stable frequency variations in the mid-to-high frequency range, making them particularly suitable for analyzing long-term sound changes. In contrast, transient events such as sheep bleating and coughing manifest differently in the CQT. Sheep bleating appears as a relatively stable low-frequency sound, while coughing exhibits sudden fluctuations covering both low and high frequencies. The Mel spectrogram is closer to human auditory perception. The energy distribution of grazing and rumination behaviors was clear and stable in the mid-frequency range, while the low-frequency components of bleating were pronounced. The sound of rapid breathing showed a uniform distribution in the mid-high frequency range, and rumination coughs exhibited distinct peaks in the high-frequency range. The STFT spectrogram was more suited to capturing short-term variations, allowing it to display characteristics of brief, high-frequency events such as bleating and coughing while eating and ruminating behaviors exhibited smooth frequency changes.

In contrast, the CQT and Mel spectrograms exhibited superior recognition capabilities when analyzing behaviors over extended time windows, such as feeding and ruminating. In comparison, the STFT spectrograms excelled at capturing transient events like bleating and coughing. Therefore, by comprehensively considering different spectrograms, we could more thoroughly extract sound features, thereby enhancing the ability to recognize sheep feeding behavior.

To further explore the intrinsic structure of the data, we employed principal component analysis (PCA) to reduce the dimensionality of high-dimensional acoustic features and visualized them into a three-dimensional feature space. The visual results of the PCA demonstrated that grazing and ruminating behaviors formed distinct clusters within the feature space, indicating significant acoustic feature distinguishability. In contrast, other sounds, such as ambient noise and the sounds of other sheep, were more diffusely distributed in the feature space, reflecting their complexity and diversity. Visualizing this feature space reveals acoustic differences between behaviors, providing valuable data for improving classification accuracy.

**Figure A1:**
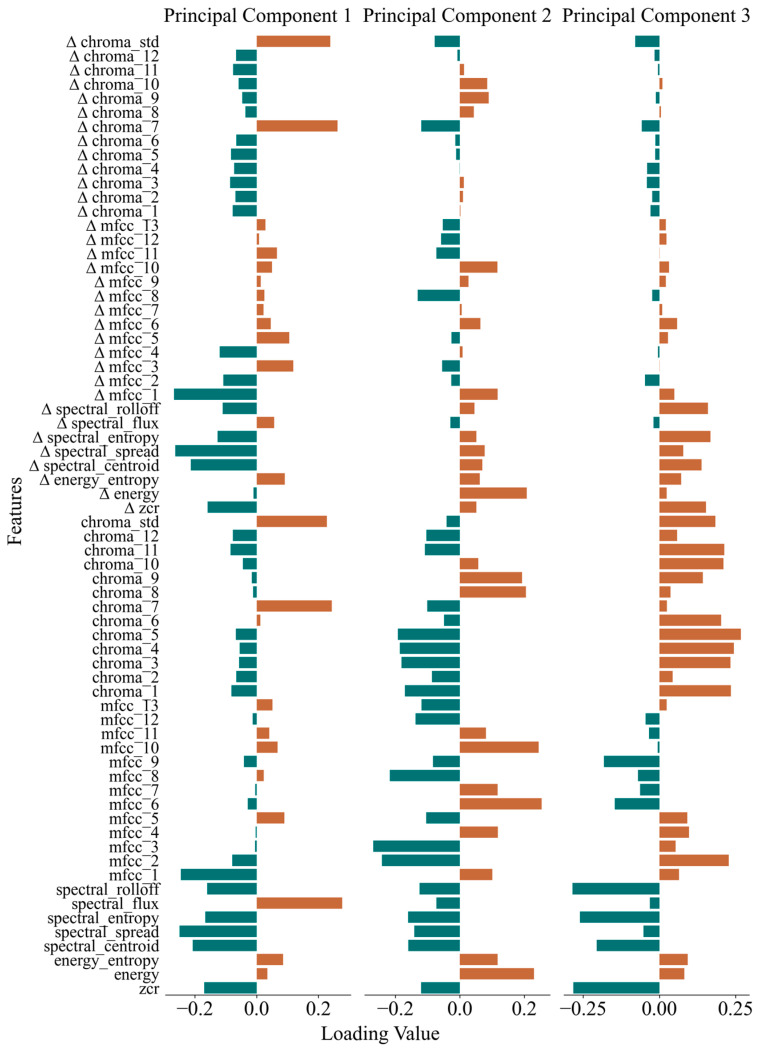
Principal component loading value analysis.

The loading values of the principal components were analyzed in depth. The results show that the feature “spectral_flux” had the highest positive loading value (0.277264) on the first principal component, which suggests that it contributed significantly to the variability of the data. In the second principal component, “mfcc_3” exhibited a significant negative loading value (−0.268651), suggesting an inverse relationship with PC2, which may reflect the role of this feature in capturing audio signal characteristics. In addition, “spectral_rolloff” had the largest negative loading value (−0.284873) on the third principal component, emphasizing its importance in distinguishing the high-frequency components of audio signals. The results of these analyses further deepen our understanding of how features affect principal components. In order to better demonstrate the results of the loading value analysis of the principal components, the relevant graphs have been placed in Appendix A, labeled Figure A1.

Through an in-depth analysis of this dataset, we have identified a significant distinction between feeding and rumination behaviors regarding acoustic features and spectrograms, thereby laying a solid foundation for the subsequent development of targeted convolutional neural networks.

### 2.4. Proposed Method

**Figure 5:**
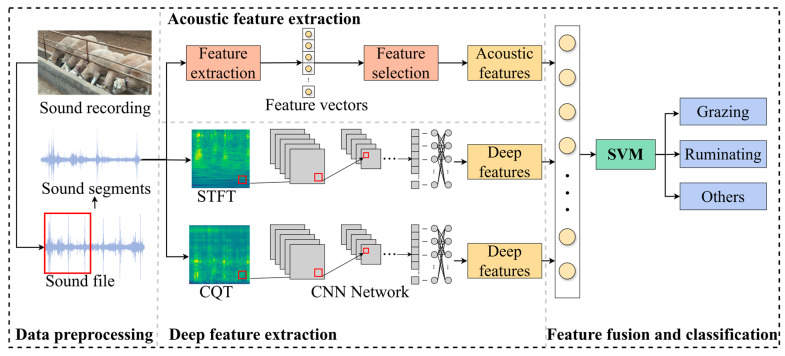
The methodology we have put forth.

When sheep graze, they often produce distinctive sounds. Thus, their feeding behavior can be identified through auditory signals. We have collected the auditory signals sheep produce during feeding and enhanced the recognition performance of sheep feeding behavior through early fusion techniques. This research introduces a new method for automatically recognizing sheep feeding behavior by extracting features from different sound representations. Figure 5 below illustrates the complete process of the proposed method.

First and foremost, extract the representative acoustic features of sheep feeding behavior from the preprocessed audio segments. Subsequently, a spectrogram is generated from the audio signal, and a lightweight shallow convolutional neural network is employed to extract deep features from the spectrogram, which reflect the characteristics of the sound in both frequency and temporal dimensions. Subsequently, through early fusion, the extracted acoustic features are integrated with the deep features of the spectrogram, forming distinctive and representative multi-domain fusion features. Finally, sheep feeding behavior is identified using a support vector machine.

#### 2.4.1. Acoustic Features

This study used the pyAudioAnalysis library for audio processing [17]. Each audio segment in the dataset served as input for extracting 68 acoustic features. Subsequently, the recursive feature elimination algorithm was employed to filter these features, ultimately identifying the key features that can represent the sound signals within the sheep pen. By integrating a time-domain analysis, frequency-domain analysis, and cepstral-domain analysis, we could comprehensively capture and reveal the characteristics of sounds within the sheepfold, providing a solid foundation for the automated detection and identification of sheep feeding behaviors.

In the time-domain analysis, we elucidated the temporal characteristics of sheep feeding sounds by calculating statistical features such as zero-crossing rate and energy. The frequency-domain analysis uncovered how feeding sounds distribute energy across various frequency components, offering essential insights into these components and their ranges. The cepstrum analysis, a more specialized approach, was employed to extract detailed acoustic features from sound signals. It provided the acoustic characteristics of sounds within the sheep pen, effectively distinguishing different types of sound signals.

By conducting an integrated analysis of time-domain, frequency-domain, and cepstral-domain features, we could deeply understand the acoustic patterns generated by sheep during feeding. These features encompassed various crucial information extracted from one-dimensional audio signals. Specifically, the time-domain features included zero-crossing rate (ZCR), energy, and energy entropy. In contrast, the frequency-domain features encompassed spectral centroid, spread, flux, roll-off, and entropy. Moreover, we also extracted 13 MFCC cepstral features and 13 chroma features. To further enhance the representation capability of features, we calculated the first-order differential features of the 38 features above, ultimately extracting 68 acoustic features. By analyzing these characteristics, we could better understand the auditory signals in the sheepfold, especially those related to sheep feeding behavior.

#### 2.4.2. Generation of Spectrograms

A spectrogram provides a two-dimensional visualization of an audio signal, mapping its frequency content over time and showing how the sound’s frequency profile evolves. In a sheep pen environment, various sounds generate distinct spectrogram patterns. Therefore, spectrograms can effectively distinguish multiple audio signals. In light of this observation, we suggest a practical method that includes producing spectrogram representations of audio signals, applying convolutional neural networks to derive intricate features from the spectrograms, and subsequently inputting these features into a support vector machine for classification tasks. The system successfully detected feeding behavior without contact. This approach is efficient and possesses practical application value, offering new technological support for the development of intelligent farming.

In this study, we employed a variety of techniques to transform audio signals from the time domain to the time–frequency domain, including the Short-Time Fourier Transform (STFT), Constant Q Transform (CQT), and Mel Spectrogram (MEL). Each method has unique advantages for capturing the auditory characteristics of sheep feeding behavior.

The Short-Time Fourier Transform is the most commonly used method for time–frequency domain conversion [18]. Segmenting the audio signal into a series of overlapping short-time windows and applying a Fourier transform to each window captures the frequency characteristics of the signal as they change over time. The formula for calculating the STFT is

(1) $$X(\tau ,k)=\sum_{n=0}^{N-1}x\left(n\right)\times w(n-\tau )\times e^{-j\frac{2\pi \mathrm{kn}}{N}}$$

Among them, x(n) denotes the audio signal, w(n−τ) is the window function, and $e^{-j\frac{2\pi \mathrm{kn}}{N}}$ constitutes the core element of the Fourier transform, used for converting time-domain signals to frequency-domain signals, where k represents the frequency. The STFT offers a combined representation of time and frequency. Still, its inherent drawback lies in the trade-off between time and frequency resolution: a shorter time window provides better time resolution but worse frequency resolution, and vice versa. In this article, N was set to 2048.

This paper employs a Constant Q transformation to handle signals with significant frequency variations more effectively. The CQT provides a more flexible signal analysis by adjusting the Q value of each frequency bandwidth, with frequency resolution varying across frequencies [19]. The CQT exhibits superior frequency resolution in the low-frequency range, while in the high-frequency range, it demonstrates superior temporal resolution. The fundamental formula of the CQT is

(2) $$X^{\mathrm{CQT}}(k)=\frac{1}{N_{k}}\sum_{n=0}^{N_{k}-1}x{(n)w}_{N_{k}}{(n)e}^{-j\frac{2\pi Q}{N_{k}}n}$$

Among them, $X^{\mathrm{CQT}}\left(k\right)$ denotes the value of the CQT coefficient at frequency k, and Q serves as the quality factor, balancing between frequency resolution and time resolution. In this article, the total frequency bin count was 200, and the frequency per octave bin count was 32, covering a total of 6.25 octaves, with the lowest frequency set at 22.05 Hz.

Additionally, the Mel spectrogram maps frequency components onto the Mel scale by emulating human auditory perception, thereby more accurately reflecting the differences in human perception of various frequencies. The computational process begins with converting the STFT of the audio signal into a spectrum, then mapping the frequencies to the Mel scale. The transformation formula for the Mel spectrum is

(3) $$\mathrm{Mel}(f)=2595\times \log_{10}\left(1+\frac{f}{700}\right)$$

Among them, f denotes the linear frequency, and Mel(f) corresponds to the respective Mel frequency. The Mel spectrum accentuates the human ear’s sensitivity to various frequency ranges, thus finding extensive application in speech recognition and music analysis. A comparison between the CQT spectrogram, Mel spectrogram, and STFT spectrogram is shown in Figure 4.

#### 2.4.3. Feature Selection

Feature selection is crucial when handling high-dimensional feature data. This approach reduces data dimensionality, decreasing computational complexity, while improving the model’s performance and ability to generalize [20]. Selecting the best features helps eliminate redundant ones, reduce noise, and improve classification accuracy. Furthermore, feature selection also aids in better understanding the critical factors behind the data, making the model more interpretable. Especially in the case of high-dimensional datasets [21], feature selection can significantly reduce the risk of overfitting, making the model more robust in practical applications.

Support vector machine recursive feature elimination (SVM-RFECV) is a feature selection technique that combines a support vector machine (SVM) and recursive feature elimination (RFE) [22]. The approach works by iteratively removing unnecessary or redundant features, which helps to minimize noise and continuously refine the feature set. In each iteration, SVM-RFECV utilizes the SVM model’s decision function to evaluate the importance of features, removing those with the most negligible impact on model performance until the most representative subset of features is selected. This approach improves model accuracy and generalization. The SVM technique is renowned for handling high-dimensional data and detecting complex patterns. When integrated with recursive feature elimination (RFE), SVM-RFECV is highly effective at processing large-scale feature sets, further enhancing the efficiency of feature selection.

In identifying sheep feeding behavior based on sound, SVM-RFECV can select the most representative acoustic features from the sheepfold audio data, thereby enhancing the accuracy of behavior classification. This method eliminates insignificant features through recursion, aiding in identifying critical acoustic features related to feeding behavior. In each iteration, SVM-RFECV assesses the contribution of features based on the F1-score to remove features that are irrelevant or have minimal impact on behavior classification and retain features that are highly relevant to behavior classification. This iterative optimization improves model accuracy in complex audio environments, providing an effective solution for identifying sheep feeding behavior.

The combination of features with the highest score is selected as the optimal set, resulting in a refined group of acoustic features. Optimized features enhance the model’s predictive accuracy and bolster its generalization capabilities in practical applications, rendering the sheep feeding behavior recognition system more precise and efficient.

#### 2.4.4. Model Construction and Training

The deep features of spectrograms are derived from audio spectrograms using deep learning. They are widely applied in speech recognition [23], sentiment analysis [24], audio classification [25], environmental sound detection [26], as well as heart sound analysis in the medical field [27], and electroencephalogram signal analysis [28,29], among others. This research utilized a lightweight, shallow convolutional neural network (CNN) model to extract deep features from spectrograms. The extracted features captured key frequency and temporal patterns, aiding in the accurate identification of sheep feeding behavior.

Our methodology employed audio spectrograms with two key driving factors. First and foremost, spectrograms can reveal sound characteristics in both frequency and time dimensions, capturing the subtle nuances between different types of sounds [23]. This characteristic has been confirmed in the automatic detection of sheep feeding behavior. Furthermore, the shallow CNN architecture effectively extracted features from spectrograms [15]. Combined with SVM classifiers, it produced highly accurate classification results and significantly enhanced execution speed. This combination effectively improved the system’s overall performance, providing a more efficient solution for real-time applications.

Our method first generated a spectrogram of the audio file and then utilized CNNs to extract the deep features of the spectrogram. Finally, these features were input into an SVM classifier for analysis. This method provided an exhaustive and robust analysis of sheep feeding events, ensuring high accuracy and reliability in identifying feeding behaviors. Through this procedure, we could effectively capture and analyze the critical auditory characteristics of sheep during feeding, thereby achieving precise behavior recognition.

**Figure 6:**
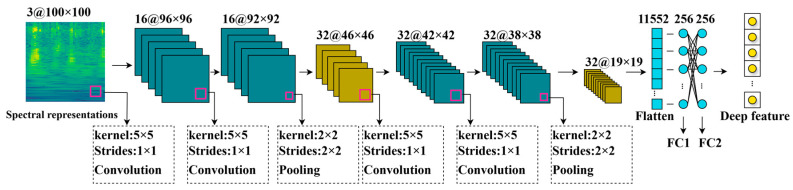
SheepVGG-Lite architecture design.

This study employed multiple convolutional neural network (CNN) architectures to extract deep features from spectrograms, including ResNet [30], Inception [31], MobileNet [32,33], EfficientNet [34], and VGG [35] series. The ResNet family of models (e.g., ResNet18, ResNet34, ResNet50) mitigates the vanishing gradient issue in deep neural networks through residual connections, enhancing their suitability for a wide range of complex tasks. Inception_v3 effectively balances performance and computational efficiency by capturing rich feature patterns through multi-scale convolution kernels. MobileNetV3 and MobileNetV4 are specifically designed for mobile devices and embedded systems, balancing lightness and efficiency, and are suitable for resource-constrained scenarios. EfficientNet has achieved a balance between computational efficiency and performance through the method of compound scaling. VGG series (such as VGG16 and VGG19) perform excellently in high-precision tasks but have significant computational overhead. Although these architectures perform well in feature extraction, they still require a balance between performance, computational cost, and model size in environments with limited datasets and resource-constrained practical deployments. It is challenging to fully meet the requirements of real-time performance and resource efficiency. In response to these challenges, we introduced a lightweight CNN architecture called SheepVGG-Lite, tailored for automatically detecting sheep feeding behavior. The feature extraction network we proposed is shown in Figure 6.

This architecture draws inspiration from VGG network design principles and simplifies the hierarchical structure to achieve efficient feature extraction with limited resources. SheepVGG-Lite combines multiple convolutional, activation, pooling, and fully connected layers. With a 3-channel spectrogram as input, it first passes through two sets of convolutional layers. The first set maps it to 16 channels, while the second set further maps it to 32 channels. After each set of convolutional layers, the ReLU activation function is applied to improve nonlinear representation, while 2 × 2 max-pooling layers help decrease the feature maps’ spatial dimensions. After the convolution and pooling processes are finished, the three-dimensional feature map is transformed into a one-dimensional vector and then processed through two fully connected layers. In subsequent experiments, the output of the first fully connected layer is denoted as FC1, and the output of the second fully connected layer is denoted as FC2. While maintaining efficient feature extraction capabilities, it significantly reduces the computational costs of the model, enabling accurate sheep feeding behavior recognition even with limited computational resources. This design is particularly well suited for real-time deployment in intelligent farming scenarios.

Since the spectrogram consisted of three-channel, two-dimensional images in this study, we utilized 2D convolutional layers to extract deep features. This approach effectively captured the local characteristics of the spectrogram across both temporal and frequency dimensions, offering a more detailed feature representation. For improved training efficiency, we chose the ReLU (Rectified Linear Unit) as the activation function, which is known for its simplicity and rapid convergence. Additionally, ReLU helps prevent the vanishing gradient issue, ensuring the stable training of deep networks. We adopted the Adam optimizer for model optimization, which integrates momentum and adaptive learning rate adjustments to enable faster and more stable convergence in various situations. The cross-entropy loss function was used in the classification tasks to evaluate the difference between the model’s predictions and the actual labels. To maintain a balance between precision and recall, especially when dealing with imbalanced data, the F1-score was selected as the primary evaluation metric.

First and foremost, we employed SheepVGG-Lite to assess the representative capacity of various spectrograms, thereby selecting the one most suited for feature extraction. Building upon this foundation, we further evaluated the performance of several commonly used convolutional neural network architectures in deep feature extraction, with particular attention to the behavior of the fully connected layers in this context. By meticulously evaluating these networks, we identified the most suitable architecture for in-depth feature extraction from spectrograms. The extracted features were then applied to subsequent classification tasks, thereby enhancing the accuracy and reliability of the model in recognizing sheep feeding behaviors.

#### 2.4.5. Feature Fusion Methods

Audio data yield acoustic features, while visual representations of sound in the form of spectrograms provide deep features, and these were combined to form a comprehensive set of representative characteristics. This fusion aimed to transcend the performance bottleneck in classifying sheep feeding behaviors, thereby achieving accurate automatic recognition. We adopted an early fusion [36] strategy, a commonly used approach, and proposed three distinct fusion methods to assess the performance of sheep feeding behavior recognition. Initially, acoustic features were derived from the audio signal, while deep features were obtained through the fully connected layers of the CNN. Next, feature extraction was achieved through the fully connected layers using two types of spectrograms as inputs, thereby accomplishing layer fusion; subsequently, inter-spectrogram fusion was attained by linking the features extracted from a particular fully connected layer of different spectrograms; finally, by combining acoustic features with the fusion mentioned above features, multi-source feature fusion was constructed. In the final step, the integrated features were fed into the SVM classifier to generate the classification results for sheep feeding behavior, as depicted in Figure 4.

### 2.5. Classification and Metrics

In identifying sheep feeding behaviors, selecting an appropriate classifier is crucial for effectively processing extracted features. We utilized early fusion methods to extract complex, high-dimensional feature vectors that encapsulated the critical characteristics of sheep feeding sounds across various domains. These vectors were subsequently merged and input into the classifier. Considering the difficulties in handling high-dimensional feature spaces and complex nonlinear decision boundaries, SVM classifiers presented a favorable choice [37]. This approach leveraged SVM’s strength in high-dimensional, nonlinear problems, improving detection accuracy and robustness.

The SVM classifier has demonstrated significant superiority in addressing the complexities of identifying sheep feeding behaviors. It demonstrates exceptional proficiency in handling high-dimensional feature spaces and effectively constructing nonlinear decision boundaries. By leveraging these capabilities, SVM classifiers can accurately detect sheep-feeding events, even amidst complex and overlapping patterns. Using SVM classifiers improved the system’s performance and reliability, advancing automatic sheep feeding behavior recognition.

We split the dataset into 80% for training and 20% for testing, ensuring no shared samples between the two subsets. The training process was conducted in two stages. In step one, we employed grid search with 5-fold cross-validation on the training set to determine the optimal combination of hyperparameters. In the subsequent step, the best hyperparameters obtained from the prior phase were utilized to retrain the model using the entire training dataset, followed by an assessment of the test set. Using grid search, we efficiently determined the optimal set of hyperparameters for the model. During the grid search, linear functions and radial basis functions were selected as kernel functions, and the C value was set to 0.1, 1 and 10.

Upon the completion of model training, we utilized the test set to evaluate its classification performance. The metrics for evaluation comprise accuracy, recall, precision, and F1-score, which are detailed in Formulas (4)–(7). The following outlines the methods used to compute these evaluation metrics:

(4) $$\mathrm{Accuracy}=\frac{\mathrm{TP}+\mathrm{TN}}{\mathrm{TP}+\mathrm{TN}+\mathrm{FP}+\mathrm{FN}}$$

(5) $$\mathrm{Recall}=\frac{\mathrm{TP}}{\mathrm{TP}+\mathrm{FN}}$$

(6) $$\mathrm{Precision}=\frac{\mathrm{TP}}{\mathrm{TP}+\mathrm{FP}}$$

(7) $$F1-\mathrm{Score}=2\;\times \frac{\mathrm{Precision}\;\times \;\mathrm{Recall}}{\mathrm{Precision}+\mathrm{Recall}}$$

## 3. Results

In this section, we elucidate the implementation methods for each experiment, delineate the primary execution environment configurations, and present the resultant findings.

### 3.1. Implementation

This research was conducted on a computer with 16 GB of memory, an AMD Ryzen 5 5600H processor, and an NVIDIA GeForce RTX 3050 Laptop GPU, operating on the Microsoft Windows 11 Home Chinese version. The entire implementation process is based on the Python (3.8.19) programming language, utilizing a variety of open-source libraries and tools. The audio processing utilized the libraries Torchaudio, pyAudioAnalysis, and Librosa [38], along with deep learning models.

The classification task used the PyTorch [39] framework, with the SVM algorithm sourced from the scikit-learn [40] library. Feature fusion was performed with the NumPy library, and the Matplotlib and Seaborn libraries handled data visualization. Comprehensive details regarding the experimental platform are provided in Table 1.

The audio data utilized in the experiment were preprocessed using the Torchaudio library, with the sampling rate set at 48,000 Hz to ensure the utmost audio quality. We extracted 68 acoustic features from audio using the pyAudioAnalysis and Librosa libraries, including the zero-crossing rate, energy, and Mel-frequency cepstral coefficients (MFCCs). The extracted features underwent standardization using the StandardScaler function from the scikit-learn library to ensure that different features were analyzed and classified on the same scale.

The extracted features were filtered during the feature selection phase using the SVM-RFECV algorithm from the scikit-learn library. The dataset was partitioned into training and test sets in an 8:2 ratio using the train_test_split function. For the classification task, SVM was chosen as the primary classification model. The SVM employed the radial basis function (RBF) as its kernel, with a penalty parameter (C) of 1.0 and Gamma configured to “auto”. In the classification experiments, the concatenate function from the NumPy library was employed to amalgamate features from diverse sources, thereby enhancing the performance of the classification model.

In order to further enhance the accuracy of sheep feeding behavior recognition, this study also conducted deep feature extraction based on spectrogram data. First, we used the Librosa library to convert audio data into a spectrogram and adjust the resulting spectrogram to 100×100 pixels. Subsequently, various convolutional neural network (CNN) models were designed and trained using the PyTorch framework. The selected models included the ResNet series (resnet18, resnet34, resnet50, resnet101, resnet152), the MobileNet series (mobilenetv3_small, mobilenetv3_large, mobilenetv4_small, mobilenetv4_medium, mobilenetv4_hybrid_medium, mobilenetv4_large), the EfficientNet series (efficientnet_b0, efficientnetv2_b0), as well as the Inception v3, VGG16, and VGG19 models.

The optimizer employed the Adam algorithm during the model training process, with an initial learning rate set at 0.001. A linear learning rate adjustment strategy was used throughout the training, culminating in a final learning rate of 0.0001. Training was configured to run for a maximum of 50 epochs, with a batch size of 32. In order to assess the model’s performance, we allocated 25% of the training data as a validation set, allowing for continuous monitoring of overfitting throughout the training process. Upon the completion of training, the deep features of the spectrogram were extracted via a fully connected layer, and subsequently, classification was performed using an SVM.

To better understand the decision-making process of the convolutional neural network model, this study applied the EigenCAM method for the interpretability analysis. This approach visualized the CNN’s activation maps, helping to identify the network’s focus areas when classifying sheep feeding behavior. Furthermore, all experimental results were visualized using the Matplotlib and Seaborn libraries. This visualization process led to various charts, including feature importance and classification results, which intuitively illustrate the experimental method and the model’s performance.

### 3.2. Evaluation of Acoustic Features

This study applied the SVM-RFECV algorithm to identify the most compelling feature subset from 68 acoustic features extracted from sheep pen audio recordings. The chosen features were subsequently input into the SVM classifier to evaluate the classification performance of these acoustic feature sets in detecting sheep feeding behavior. The SVM-RFECV employed an iterative approach to eliminate unnecessary or redundant features, effectively reducing noise interference and enhancing the model’s robustness. Furthermore, the SVM-RFECV harnessed the robust capabilities of support vector machines (SVMs), renowned for their effectiveness in handling high-dimensional data and identifying complex patterns. Integrating a SVM and RFECV provided precise feature selection while ensuring computational efficiency. For reliable evaluation results, we employed a five-fold cross-validation approach.

**Figure 7:**
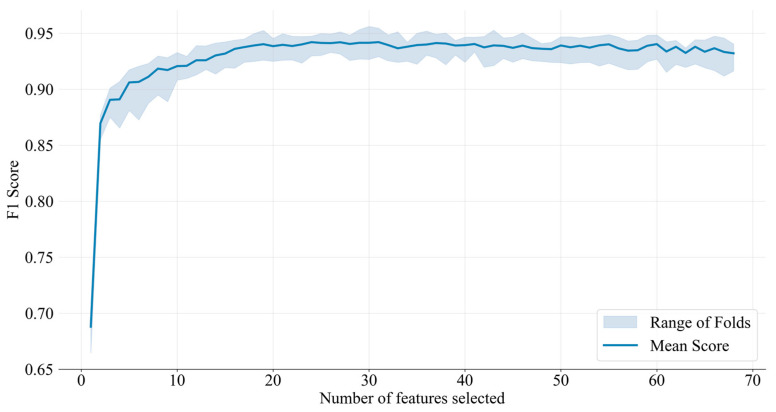
Evaluation outcomes of acoustic features using five-fold cross-validation.

We utilized the permutation feature importance method for feature selection, allowing for an unbiased evaluation of each feature’s contribution to accurate classification. In the process of SVM-RFECV feature selection, we assessed the number of selected features along with their classification performance using cross-validation scores. The evaluation outcomes based on five-fold cross-validation are depicted in Figure 7. According to Figure 7, the F1-score reached a stable value when the feature set included 19 attributes. As the feature count increased, a gradual improvement in the model’s accuracy could be observed. The model attained its best evaluation metrics when 31 features were used, achieving an accuracy of 94.30%, a precision of 94.34%, a recall of 94.30%, and an F1-score of 94.31%.

### 3.3. Evaluation of Feature Extraction Capabilities of Shallow CNN

This study seeks to assess the effectiveness of a shallow CNN feature extractor in generating deep features from spectrograms in the provided dataset. Initially, the audio data were transformed into three types of spectrograms: MEL, STFT, and CQT. Subsequently, a custom CNN feature extractor was employed to extract deep features, which were then input into an SVM classifier to assess the performance of these features, particularly from the fully connected layers (FC1) and (FC2). At the same time, the spectrogram with the highest performance based on evaluation metrics was selected as the default for future experiments, enabling comparisons across different feature extraction methods.

In this experiment, the shallow CNN feature extractor’s capability to extract deep features under various spectrograms was evaluated, with the results presented in Table 2. According to the analysis results, the FC1 layer of the MEL spectrogram performed the best on the SVM classifier, achieving an accuracy of 94.3% and a precision of 94.38%, with an F1-score of 94.31. The FC2 layer of the MEL spectrogram subsequently attained a notable accuracy of 93.61%, along with an F1-score of 93.61%. The CQT spectrogram’s FC1 layer also performed admirably, with an accuracy rate of 93.73% and an F1-score of 93.74%.

### 3.4. Comparative Study of Shallow CNN Networks and Deep Neural Network Models Under Transfer Learning

Transfer learning refers to the process in which a model with extensive labels and pre-existing knowledge is leveraged and applied to the domain of the target learning task. In this learning process, the feature extraction by deep neural networks progresses from the simple to the intricate, advancing from the general characteristics such as the contours and lines of images to higher-order abstract features. Considering this, the study preserves the lower-layer parameters of the pre-trained model during transfer learning, while only adjusting the higher-layer weights to fine-tune the deep model. This approach leverages the pre-trained model’s general features, reducing re-learning time and speeding up training.

To compare the impact of feature extraction by networks of varying depths on classification performance, we conducted an experiment that evaluated ResNet18, ResNet34, ResNet50, ResNet101, ResNet152, Inception_v3, MobileNetV3_small, MobileNetV3_large, MobileNetV4_small, MobileNetV4_medium, MobileNetV4_hybrid_medium, MobileNetV4_large, EfficientNet_B0, EfficientNetV2_B0, VGG16, VGG19, and other models in extracting deep features from spectrograms. Spectrogram representations of audio segments from the sheep barn were used as input, with the most suitable CNN chosen as the feature extractor. An SVM classifier was then employed to identify sheep feeding behaviors.

Before feature extraction, it was essential to construct the model and load the pre-trained weights from Hugging Face and Timm. We adapted the pre-trained model by removing and replacing the original classifier with a custom classifier designed explicitly for our task. This custom classifier included a fully connected layer with 1000 neurons and an output layer with three neurons. During the transfer learning process, we froze the convolutional layer parameters of the model, fine-tuning only the higher-level weights. This strategy effectively leveraged the generalization capability of the pre-trained model, reducing the time required for retraining and expediting the training speed for the target task.

We employed a fine-tuned network during the feature extraction process, retraining the model’s fully connected layer and final classification layer based on the dataset we constructed. The model initialization parameters were based on ImageNet pre-trained weights. The input image was a three-channel RGB image. The chosen network determined the size of the image, and the image data were normalized. All networks retained their original feature extraction parts. After deep feature extraction, the output of the last fully connected layer 1000 features was input into the SVM to complete the classification task of sheep feeding behavior.

The experimental results presented in Table 3 highlight the varying performances of different deep neural network models in feature extraction and classification tasks. Overall, the SheepVGG-Lite model demonstrated superior performance across all evaluation metrics, achieving an accuracy, recall, precision, and F1-score of 93.73%. This performance represents the current state-of-the-art model, particularly well suited for identifying sheep feeding behavior within a pen, as it excelled across all evaluation metrics. The runner-up model was MobileNetV3 (large). It demonstrated an accuracy, recall rate, precision, and F1-score of 92.70%, showcasing an exceptionally balanced performance. This balanced performance makes it well suited for applications that demand both efficiency and high performance.

Moreover, the performances of VGG16 and EfficientNetV2 B0 were also relatively commendable. Particularly notable was VGG16, which boasted an impressive accuracy rate of 91.79%, with all metrics approaching 92%. Meanwhile, EfficientNetV2 B0 also delivered a commendable performance with an accuracy rate of 91.11%.

Nevertheless, specific models exhibited subpar performance in this task. For instance, the accuracy, recall, precision, and F1-score of models such as MobileNetV4-Conv-Large, MobileNetV4-Conv-Medium, MobileNetV4-Hybrid-Medium, and ResNet101 hovered around 80%, which was significantly lower than that of the optimal models. Among them, the accuracy of MobileNetV4-Conv-Large was merely 79.93%.

Specifically, among the ResNet series models, ResNet18 exhibited the best performance, achieving an accuracy rate of 89.38%. However, with the increasing network depth (ResNet34, ResNet50, ResNet101, ResNet152), accuracy and other metrics did not significantly improve and even declined. This decline could be attributed to overfitting caused by the increased complexity of the model. The performance of Inception v3 was satisfactory, with an accuracy rate of 87.80%, which was above average. The performance of the MobileNet series was quite uneven, with MobileNetV3 (large) being the most outstanding, while the MobileNetV4 series models did not performed well in this task. In the EfficientNet series, particularly EfficientNetV2 B0, the performance was awe-inspiring, achieving an accuracy of 91.11%. It stands as a model worth serious consideration. Within the VGG series, both VGG16 and VGG19 exhibited commendable performances, particularly VGG16, which boasted an accuracy of 91.79%.

Considering the comprehensive performance of each model, SheepVGG-Lite emerges was the optimal choice, excelling in all metrics.

### 3.5. Evaluation of Feature Fusion

This experiment assessed how various fusion algorithms affect the classification performance in recognizing sheep feeding behavior and determined the most effective fusion strategy. The results of the experiment are presented in Table 4. Under the condition of single spectrogram input, the MEL spectrogram’s FC1 and FC2 layers were chosen for fusion research. The findings revealed that post-fusion classification accuracy reached 93.85%. Under the dual spectrogram input, based on the MEL spectrogram of FC1, the STFT spectrogram of FC1 and FC2, and the CQT spectrogram of FC1, a fusion study of the spectrograms was conducted, revealing that the MEL spectrogram of FC1 and CQT spectrogram of FC1 achieved a classification accuracy improvement to 95.45%; when the STFT spectrogram of FC1 and CQT of FC1 were fused, the accuracy further increased to 95.67%.

Finally, 31 optimal acoustic features were selected and integrated with the best spectrogram features. The integration resulted in a notable enhancement in classification performance, particularly when acoustic features were combined with the FC1 layers of the spectrogram and the CQT, leading to a classification accuracy of 96.47%. These findings indicate that combining the acoustic features of sound signals with the deep features in spectrogram representations can significantly enhance the accuracy of sheep feeding behavior recognition.

In conclusion, the experimental findings confirm the advantage of integrated features for accurately identifying sheep feeding behavior. Using the SVM-RFECV algorithm to extract acoustic features, in combination with various spectrogram feature fusion strategies, resulted in solid classification performance, further validating the effectiveness and practicality of the proposed approach.

To confirm the practical applicability of our proposed method in enhancing animal welfare, we thoroughly evaluated the model’s feature extraction speed and overall dimensions. For effective deployment, a robust classification model must demonstrate high classification performance and excel in recognition speed and model compactness, as these elements are essential for real-world applications.

**Figure 8:**
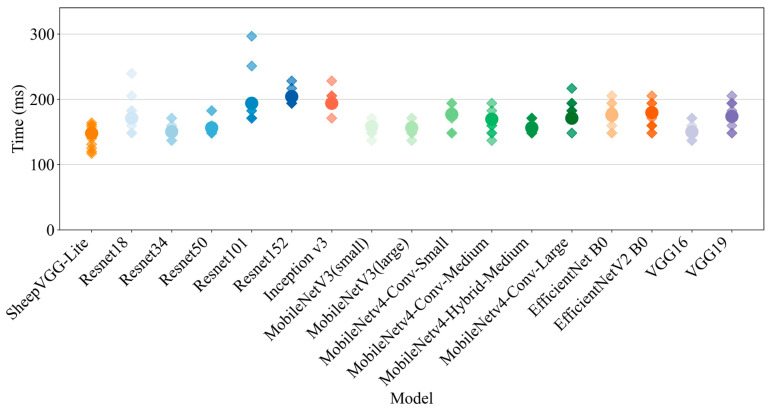
Inference time performance comparison of models.

Ten calculations were conducted on 10 test set samples using our computational platform, with no additional load. The outcomes are shown in Figure 8. The experimental results reveal that SheepVGG-Lite outperformed other models across all key performance metrics. Specifically, the custom model achieved an accuracy of 93.73% and precision and recall rates of 93.73% and 93.72%, respectively. Additionally, the model’s inference time was just 13.6 milliseconds, notably faster than other more complex models. These findings suggest that SheepVGG-Lite strikes an ideal balance between performance and efficiency.

**Figure 9:**
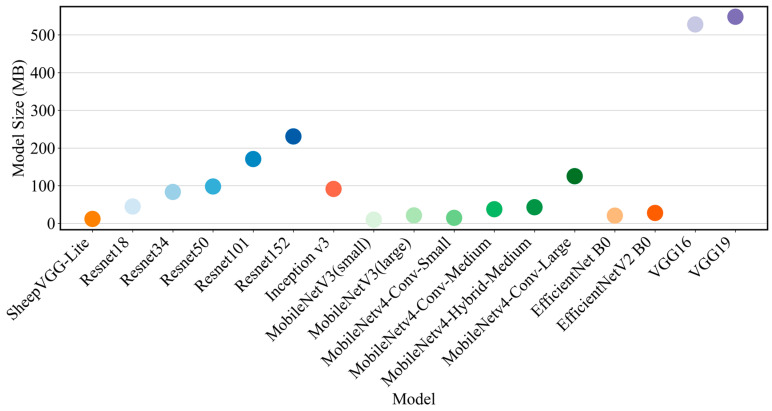
Model size performance comparison of models.

Finally, we evaluated the model size, as it directly impacts the deployment scenarios. The results are shown in Figure 9 below. Through an analysis of the model size, SheepVGG-Lite demonstrates a remarkable advantage in lightweight design, with a model size of only 11.7 MB. Additionally, it exhibits excellent comprehensive performance, making it highly suitable for resource-constrained application scenarios. In comparison, the models of MobileNetV3 (small) and MobileNetV3 (large) were 9.8 MB and 21.1 MB, respectively. They are also suitable for portable devices, but their performances were slightly inferior to SheepVGG-Lite’s. While larger models such as ResNet50, ResNet101, and ResNet152 boasted excellent performance, they are only suitable for environments with ample resources. In summary, SheepVGG-Lite, with its lightweight design and high performance, has become the preferred choice in environments demanding high efficiency.

Compared with other models, SheepVGG-Lite excels in both performance metrics and model efficiency, making it the most optimal model. Although different models, such as ResNet18 and MobileNetV3_Large, demonstrate certain competitiveness in specific scenarios, after a comprehensive consideration of accuracy, inference time, and model size, SheepVGG-Lite is undoubtedly the optimal choice, particularly suited for applications demanding high performance and efficiency.

### 3.6. Analysis of Model Interpretability

Accurately understanding the model’s focus areas for different behaviors in sheep behavior recognition is crucial to optimizing model performance. By visualizing the internal characteristics of the model, we can better elucidate the model’s decision-making process and verify its accuracy and reliability in detecting specific behaviors. To this end, we employed a visual methodology to assess the model’s responsiveness to sheep’s feeding, ruminating, and bleating behaviors and the feature activations of these behaviors within the model. Our ultimate goal is to utilize these visualized results to assess the model’s efficacy further and enhance its capability in behavior classification.

In this experiment, we employed the EigenCAM [38] visualization technique to elucidate the internal feature activations of the model. EigenCAM is a feature map-based visualization technique that aids in understanding how the model makes classification decisions on input data by calculating the primary activation regions of the convolutional layer outputs. Compared with other methods, EigenCAM can distinctly reveal the focus areas of the model in the time–frequency domain, making it especially suitable for complex input data such as spectrograms. As illustrated in the figure, EigenCAM has successfully demonstrated the activation characteristics of the model when processing different behaviors such as eating, ruminating, bleating, panting, and drinking water. The visualization results of eating behaviors reveal that the model simultaneously focuses on both the low-frequency and high-frequency parts of the spectrogram, which aligns with the characteristic sounds produced during chewing.

**Figure 10:**
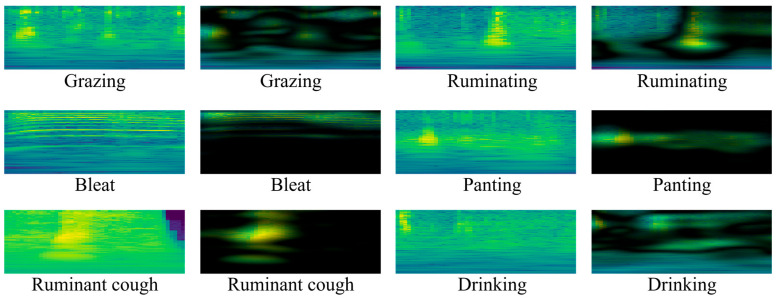
Visualization of spectrogram feature activation for different sheep behaviors using EigenCAM.

Regarding ruminative behavior, the model similarly exhibited significant attention to low-frequency features, indicating that the model can effectively capture the rhythmic characteristics of ruminating at low frequencies. In the behaviors of bleating and panting, the model exhibited a solid response to the more prominent transient high-frequency events in the spectrogram. These results indicate that the model can distinguish brief sound events, such as bleating or panting. In the spectrograms of behaviors such as drinking water and ruminating coughs, EigenCAM likewise demonstrates the model’s commendable sensitivity to the distinctive characteristics of these behaviors. These visual results show that the model exhibits consistent and reasonable activation patterns when processing different sheep feeding behaviors. These observations indicate that the model can classify behaviors with remarkable accuracy based on the features of the input spectrogram (Figure 10).

## 4. Discussion

This research significantly enhances the classification performance of sheep feeding behavior through multi-domain feature fusion, especially under bispectral fusion, achieving a classification accuracy of 96.47%. Compared with traditional methods applied in controlled environments (e.g., the RNN model achieving 96.13% accuracy [12]), this study uses data collected in real production environments with high noise interference. Although studies using IMU sensors at multiple positions (jaw, neck, and hind leg) have achieved up to 99.3% accuracy, the practicality of attaching sensors at multiple points is limited in real-world settings. Their performance with a single sensor is lower than our approach, dropping to 81.7% when only the jaw sensor is used [6]. This result indicates that different representations of audio signals hold immense potential in complex behavior recognition tasks and have significant reference values in practical applications. Additionally, high classification accuracy enables feeding personnel to adjust feed distribution based on each sheep’s feeding patterns, thereby achieving personalized feeding, promoting healthy growth, and improving production efficiency. Furthermore, by identifying the impact of different feeds on feeding behavior, this system assists in optimizing feed choices, reducing waste, and lowering feeding costs.

The feature selection results of this study indicate that in the sheep feeding behavior classification task, the SVM-RFECV algorithm selected 31 optimal features from 68 acoustic features. These features include time-domain features, frequency-domain features, MFCC, Chroma features, and their delta features. Compared with Shen’s research [36], this study, while retaining core spectral features, introduced Chroma and delta features better to describe the dynamic changes in signals over time. The selection of these features helped capture the complex sound characteristics of feeding behavior, including energy fluctuations, spectral properties, and their dynamic trends, thereby enhancing the model’s discriminative ability and robustness. Although these features improved classification performance, a single feature alone could not fully capture the complexity of the behavior patterns. Therefore, this study combined these acoustic features with deep spectrogram features and further improved classification accuracy through multi-domain feature fusion.

The spectrogram’s deep features have demonstrated exceptional performance in identifying sheep feeding behavior. According to the results of Table 2, the performance of the FC1 layer of the spectrogram in the SVM classifier is the best, with an accuracy rate of 94.30%. Although FC2 performed well in the spectrogram, it was slightly inferior to FC1. These results indicate that shallow feature extraction is already sufficiently effective, and further increasing the features of the fully connected layer has a limited effect on improving classification performance. This finding is consistent with the results of Wang [15] and Shen [36].

The results from the feature fusion experiments further confirm the effectiveness of integrating features from multiple domains. The classification performance has been significantly enhanced by integrating acoustic features with deep spectrogram characteristics. Especially under the input of single and dual spectrograms, the classification accuracy after fusion was improved dramatically, particularly in the STFT spectrogram and CQT spectrogram, which achieved an FC1 fusion classification accuracy of 95.67%. Integrating 31 optimal acoustic and deep spectral features through a multi-domain feature fusion scheme achieved 96.47% classification accuracy. This result underscores the significant role of complementarity between features in enhancing model performance. This study achieved high-accuracy recognition of sheep feeding behavior through the fusion of acoustic features and deep abstract features. We believe that this approach is not only applicable to the recognition of feeding behavior in sheep but also has potential value for similar behavior recognition in other herbivorous livestock. Furthermore, since acoustic signals provide a non-invasive means of observation in wildlife behavior monitoring, the methods used in this study also offer valuable insights for detecting related behaviors in wildlife, especially in identifying behavioral patterns that can be captured through acoustic signals.

Based on these findings, future research could further explore multimodal sensor fusion for more comprehensive monitoring of sheep behavior. A bimodal scheme combining accelerometers and sound sensors can help overcome the location-dependent limitations of accelerometers in a herd environment. Accelerometers provide signals of the sheep’s movements, reflecting activity levels and positional shifts, while sound sensors record acoustic characteristics of chewing and swallowing. Fusing these two types of data will allow us to more accurately distinguish between different types of behavior, especially when distinguishing feeding from other similar actions. In addition, future research should focus on how to effectively integrate accelerometer and acoustic data to ensure that methods are cost-effective and highly maintainable. By exploring the potential of multi-sensor fusion, we expect to achieve comprehensive monitoring of animal behavior in herd environments and provide strong technical support for livestock management and animal welfare.

Furthermore, the results in Table 4 indicate that the custom model has achieved an excellent balance between accuracy and efficiency, significantly surpassing other models. Despite the model’s relatively small size and rapid processing speed, it maintains high-precision classification performance, making it suitable for deployment in natural farm environments. However, it is essential to note that converting audio to spectrograms may consume additional computational resources. Therefore, future research must thoroughly consider these potential challenges in actual deployment. Moreover, environmental noise, equipment malfunctions, and other anomalies may compromise the stability of the model. Therefore, enhancing the model’s robustness is essential to ensure its reliability in practical applications.

This study demonstrates the advantages of acoustic monitoring in sheep behavior recognition; however, certain factors in extreme weather and farm environments may still impact monitoring effectiveness. Firstly, noise from extreme weather conditions, such as strong winds and heavy rain, significantly reduces the signal-to-noise ratio. Since the sounds generated by feeding behaviors are relatively faint, noise reduction processing can weaken or even eliminate many signals of interest, making it unsuitable for the current data. In the future, we will consider enhancing the system’s adaptability in extreme environments by integrating multiple sensors, such as acoustic sensors and accelerometers. Compared with acoustic sensors, accelerometers are less sensitive to environmental noise and can provide more reliable monitoring data under adverse conditions. Additionally, we have considered the potential interference from other animals on the farm, such as birds, during data collection. Although the acoustic model accounts for common sounds in the farm environment, certain bird sounds are highly unpredictable (e.g., varying flight ranges and occasional appearances in the monitoring area), making it difficult to completely avoid such interference. Moving forward, we will further explore optimization methods to mitigate interference from other animal sounds, aiming to improve the model’s robustness.

When building deep learning models, the quality and quantity of data determine the model’s performance, especially in sheep feeding behavior recognition. A significant challenge we encounter is the requirement for extensive labeled data, a labor-intensive and expensive process. A lack of sufficient labeled data can result in weak generalization of the model. The manual annotation process is costly and introduces bias, which can negatively affect the model’s ability to generalize. The subjective judgment of annotators during manual labeling may result in certain features being over- or under-labeled, introducing selection bias that impacts the model’s performance in real-world applications. Therefore, reducing bias from manual labeling is crucial. We aim to implement automated sound detection technology in the future, allowing machines to label and classify sheep vocalizations autonomously, thereby minimizing human-induced errors. This automated labeling process is expected to significantly improve data processing efficiency, enabling large-scale data collection and annotation and ultimately laying a solid foundation for model training.

## 5. Conclusions

This study achieved significant performance improvements in the classification of sheep feeding behavior through feature fusion, particularly under bispectrum fusion, where the classification accuracy reached 96.47%, highlighting the remarkable potential of diverse data representations in complex behavior recognition. In this research, we selected 31 optimal acoustic features using the SVM-RFECV algorithm and combined them with deep spectrogram features. Additionally, we proposed a bespoke model that achieved a high balance between classification performance, processing speed, and model size, showcasing its extensive feasibility for deployment in actual farm environments. By delving into the research on multi-domain feature fusion technology, this study advances the technological progress in sheep feeding behavior recognition and lays a solid foundation for precision feeding research in sheep.

## Figures and Tables

**Table 1:** Hardware and software specifications.

Hardware/Package	Version/Details
Memory	16 GB
CPU	AMD Ryzen 5 5600H
GPU	NVIDIA GeForce RTX 3050 Laptop GPU
Operating System	Microsoft Windows 11 Home Chinese Edition
Torchaudio	2.3.0+cu121
pyAudioAnalysis	0.3.14
Librosa	0.10.2.post1
PyTorch	2.3.0+cu121
scikit-learn	1.3.2
NumPy	1.24.4
Matplotlib	3.7.5
Seaborn	0.13.2
grad-cam	1.5.2

**Table 2:** Feature extraction and evaluation of shallow CNN extractor on spectrograms.

Features	Accuracy (%)	Recall (%)	Precision (%)	F1-Score (%)
MEL Spectrogram FC1	94.30	94.30	94.38	94.31
MEL Spectrogram FC2	93.61	93.61	93.72	93.61
STFT Spectrogram FC1	92.70	92.70	92.75	91.65
STFT Spectrogram FC2	91.68	91.68	91.76	91.65
CQT Spectrogram FC1	93.73	93.73	93.76	93.74
CQT Spectrogram FC2	93.73	93.73	93.72	93.72

**Table 3:** Comparison of deep feature extraction models based on transfer learning.

Model	Accuracy (%)	Recall (%)	Precision (%)	F1-Score (%)
Resnet18	89.38	89.38	89.46	89.35
Resnet34	85.68	85.68	85.83	85.71
Resnet50	85.22	85.22	85.33	85.21
Resnet101	84.61	84.61	84.65	84.58
Resnet152	86.89	86.89	86.96	86.87
Inception v3	87.80	87.80	87.90	87.79
MobileNetV3 (small)	89.97	89.97	90.01	89.92
MobileNetV3 (large)	92.70	92.70	92.70	92.70
MobileNetv4-Conv-Small	85.75	85.75	85.88	85.78
MobileNetv4-Conv-Medium	82.21	82.21	82.36	82.24
MobileNetv4-Hybrid-Medium	83.24	83.24	83.30	83.22
MobileNetv4-Conv-Large	79.93	79.93	80.01	79.93
EfficientNet B0	89.28	89.28	89.34	89.30
EfficientNetV2 B0	91.11	91.11	91.23	91.13
VGG16	91.79	91.80	91.92	91.79
VGG19	89.85	89.85	89.99	89.86
SheepVGG-Lite	93.73	93.73	93.72	93.72

**Table 4:** Effect of various fusion strategies on the classification performance of sheep feeding behavior.

Features	Accuracy (%)	Recall (%)	Precision (%)	F1-Score (%)
MEL FC1+ MEL FC2	93.84	93.84	93.92	93.85
MEL FC1 + STFT FC1	94.64	94.64	94.70	94.64
MEL FC1 + CQT FC1	95.44	95.44	95.46	95.45
STFT FC1 + CQT FC1	95.67	95.67	95.68	95.67
Acoustic	94.30	94.34	94.30	94.31
CQT FC1 + STFT FC1 + Acoustic	96.47	96.47	96.47	96.46

## Data Availability

The data presented in this study are available from the corresponding author upon reasonable request. The data are not publicly available due to privacy and confidentiality agreements that protect the commercial interests of the farms involved.
